# Long-term effects of the *SLC2A9* G844A and *SLC22A12* C246T variants on serum uric acid concentrations in children

**DOI:** 10.1186/s12887-018-1272-y

**Published:** 2018-09-06

**Authors:** Hye Ah Lee, Bo Hyun Park, Eun Ae Park, Su Jin Cho, Hae Soon Kim, Hyesook Park

**Affiliations:** 1 Department of Preventive Medicine, College of Medicine, Ewha Womans University, 1071, Anyangcheon-ro, Yangcheon-ku, Seoul, 158-710 Korea; 2 Clinical Trial Center, Mokdong Hospital, Ewha Womans University, Seoul, Korea; 3 Department of Pediatrics, College of Medicine, Ewha Womans University, Seoul, Korea

**Keywords:** Children, Longitudinal study, Urate transporter 1, Uric acid

## Abstract

**Background:**

We evaluated the effects of two single*-*nucleotide polymorphisms on UA concentrations in the first decade of life using repeated-measures data.

**Methods:**

We included all subjects who were followed-up at least once and for whom we had both UA and genotypic data (i.e., 375, 204, 307, and 363 patients aged 3, 5, 7, and 9 years, respectively). All participated in the Ewha Birth and Growth Cohort study. We used a mixed model analysis to estimate the longitudinal association of serum UA concentration due to the rs3825017 (*SLC22A12* c. 246C > T) and rs16890979 (*SLC2A9* c. 844G > A) genotypes.

**Results:**

Overall, the tracking coefficient of UA concentrations in children 3 to 9 years of age was 0.31, and was higher in boys than in girls (0.34 vs. 0.29, respectively). Regarding individual variance, serum UA concentrations decreased as age increased (*β* = − 0.07, *p* < 0.05), but there were no significant differences by sex. The effects of rs3825017 on UA concentration were significant in boys, but not in girls. Boys with the T allele of rs3825017 had higher concentrations than their counterparts regardless of the time of follow-up. The rs16890979 genotypes were not significantly associated with serum UA concentration in either sex.

**Conclusion:**

This study showed that rs3825017 in the *SLC22A12* gene was associated with UA concentration in childhood.

## Background

Uric acid (UA) is the major endpoint of purine metabolism in humans [1, 2]. Serum UA concentration is modulated by urate excretion and reabsorption, which mainly occurs in the kidneys [1, 2]. An abnormally high serum concentration of UA is a predictor of vascular disease, gout, and renal disease [1], and is also associated with pediatric hypertension [3]. In addition, a longitudinal cohort study of the Bogalusa Heart Study reported that UA concentration in childhood is a significant predictor of blood pressure later in life [4].

It has been suggested that genetic variants markedly contribute to UA concentrations [5], with an estimated heritability of − 70% [6]. Genome-wide association (GWA) studies of common variants identified more than 30 genetic loci associated with the UA concentrations, mostly in urate transporters, such as the genes encoding solute carrier family 2 facilitated glucose transporter member 9 (also known as *SLC2A9* encoding GLUT9), solute carrier family 22 (organic anion/cation transporter) member 12 (also known as *SLC22A12* encoding URAT1), and the ATP-binding cassette transporter subfamily G member 2 (*ABCG2*) [2, 5]. The importance of URAT1 and GLUT9 for regulating blood urate levels was confirmed, and mutations in *SLC22A12* and *SLC2A9* have been reported to be responsible for hypouricemia [7]. *ABCG2* is also known to be a major cause of gout and hyperuricemia that acts by decreasing urate excretion [8]. Among these candidate genes, the missense single-nucleotide polymorphisms (SNPs) rs3733591 (c. 881G > A) and rs16890979 (c. 844G > A) in the *SLC2A9* gene had significant effects on gout in an Asian population [9]. Both polymorphisms protected against the development of gout, and rs16890979 showed a stronger association than rs3733591 [9]. The protective effect of common variant p.V12 M (rs2231137) in *ABCG2* on gout was also reported in a meta-analysis (odds ratio = 0.73, *p* < 0.0001) [10], but the functional characterization of this variant did not show altered urate transport activity [11]. As variants in *SLC22A12* related to hypouricemia, c.774G > A and c.269G > A were identified in Japanese [12] and Korean [13] subjects, and c.1245_1253del and c.1400C > T were found in the Roma population [14]. In addition, a recent study of Korean men reported five tagging SNPs of URAT1, of which rs3825017 (c. 246C > T) showed a strong association with hyperuricemia despite the small sample size [15]. Except for genetic effects, birth weight [16], dietary intake [1, 17], and body mass index (BMI) [18] are also correlated with UA concentrations.

Evidence from GWA and epidemiology studies has suggested a number of candidate genes and a recent study from the Viva La Familia Study also reported genetic variants in *SLC2A9* associated with UA concentrations in children [19]. Studies evaluating the effects of gene variants in the context of controlling UA concentrations are important, but insufficient research has been conducted in children. On reviewing the literature and considering the possibility of genotype analysis, we assessed the effects of two SNPs (rs3825017 genotype of *SLC22A12* and rs16890979 genotype of *SLC2A9*) of UA-related genes on the UA concentration in childhood using repeated-measures UA data derived from the Ewha Birth & Growth Cohort study to determine whether genetic variants influence the UA concentrations during the first 10 years of life. Because there is little data on how the UA concentration is maintained over time, we also estimated the tracking coefficient of the UA concentrations during childhood.

## Methods

### Study subjects

This study was conducted as part of the ongoing Ewha Birth & Growth Cohort study, a single-hospital-based prospective cohort that was established in 2001–2006. The cohort was started to investigate the risk and preventive factors that may be associated with growth and disease susceptibility via longitudinal observations. The study enrolled 940 subjects at 24–28 weeks pregnancy who visited Ewha Womans University Mokdong Hospital, Seoul, Korea for prenatal care. We conducted follow-up when their offspring reached the ages of 3, 5, and 7 years and annually thereafter. By 2016, the follow-up of children aged 9 years had been completed. The methodology of the cohort study has been reported elsewhere [20, 21]. In this study, two SNPs were genotyped in the subjects who were followed up at least once. There were 471, 400, 364, and 400 cohort members who were followed up at the ages of 3, 5, 7, and 9 years, respectively. Of these, we included subjects who had both UA and genotype data (i.e., 375, 204, 307, and 363 at ages 3, 5, 7, and 9 years, respectively). This study was composed of 620 children because many children attended multiple follow-up visits. Written informed consent for participation in the study was obtained from the parents or guardians of all of the study participants at the time of follow-up. The study protocol was approved by the Institutional Review Board of Ewha Womans University Hospital (approval No. EUMC 2015–04-048). All processes were performed in accordance with relevant guidelines and regulations.

### Uric acid measurements

When visiting a hospital to participate in the follow-up program, we collected a blood sample from an antecubital vein in a vacutainer tube containing ethylenediaminetetraacetic acid (EDTA). All blood sample were obtained after fasting at least 8 h. Serum UA concentrations were measured using the uricase-peroxidase coupled reaction method on a Hitachi Auto-analyzer 7600 (Hitachi, Fukuoka, Japan) in the Clinical Biochemistry Department of the Seegene Medical Foundation, Seoul, Korea. UA measurements during follow-up were performed in the same way.

### Genotyping the two SNPs

The genotypes of rs3825017 in *SLC22A12* and rs16890979 in *SLC2A9* were screened using the TaqMan fluorogenic 5′ nuclease assay (ABI, Foster City, CA, USA). The final volume of the polymerase chain reaction (PCR) was 5 μL, and each reaction contained 10 ng genomic DNA, 2.5 μL TaqMan Universal PCR Master Mix, and 0.13 μL 20× Assay Mix. The thermal cycle conditions were as follows: 50 °C for 2 min to activate the uracil N-glycosylase and to prevent carry-over contamination, 95 °C for 10 min to activate the DNA polymerase, followed by 45 cycles at 95 °C for 15 s and 60 °C for 1 min. All of the PCR reactions were performed using 384-well plates on a Dual 384-Well GeneAmp PCR System 9700 (ABI). The endpoint fluorescent readings were performed on an ABI PRISM 7900 HT Sequence Detection System (ABI). Duplicate samples and negative controls were included to ensure the accuracy of genotyping, which was performed at DNA Link Inc. in Seoul, Korea. The distribution of the two SNPs in the study subjects satisfied Hardy–Weinberg equilibrium.

### Covariates

As covariates, we considered BMI and birth weight, based on previous studies [16, 18]. Maternal education level was used as a measure of socioeconomic status because it changes little over time (graduated from high school or some college or higher). Anthropometric data were collected by trained researchers when the subjects visited the hospital to participate in the follow-up program. Height and weight were measured to the nearest 0.1 cm and 0.1 kg, respectively, while wearing light clothing and no shoes using a stadiometer and calibrated scale. BMI was calculated as weight (kg)/height^2^ (m^2^). BMI at each follow-up point was considered a covariate. Birth weight was obtained from the hospital birth records.

### Statistical analysis

Numerical variables are presented as the mean and standard deviation and categorical variables were presented as the number of subjects and percent. Using repeated-measures data for UA concentrations in subjects aged 3, 5, 7, and 9 years, we estimated the intra-class correlation (ICC) as the tracking coefficient through mixed model analysis [22]. There was no critical tracking value. We interpreted < 0.3 as weak, 0.3–0.6 as moderate, and > 0.6 as strong, based on a previous study [23]. The average difference in UA concentration by sex was evaluated using Student’s *t*-test. The average UA concentration according to genotype was analyzed using a generalized linear model.

To investigate the long-lasting effects of genetic variants on UA concentration, we used mixed model analysis with a random intercept model. Analyses were conducted using a variance component structure based on lower Akaike information criterion (AIC) values for model selection. The genetic variant used a dominant model (CC vs. CT + TT genotypes) to ensure that the groups had sufficient subjects. Maternal education level and birth weight were treated as time-independent covariates. The BMI at each follow-up visit was considered a time-dependent covariate. To assess the effect modifier, we also included the interaction between sex and genotype as a dominant model.

All of the statistical tests were conducted using SAS 9.4 (SAS Institute, Cary, NC, USA) with two-sided tests, and a *p* value < 0.05 was considered statistically significant. For the interaction, *P* values < 0.1 were considered to be significant, based on a previous report [24].

## Results

Two SNPs were examined in 620 children. The rs3825017 allele frequencies of CC, CT, and TT were 61.1%, 34.9%, and 4.0%, respectively. The rs16890979 allele frequencies of GG, GA, and AA were 99.0%, 1.0%, and 0.0%, respectively (Table 1). The genetic polymorphism distribution did not differ by sex (*p* > 0.05, χ^2^ test) and there was no sex difference in UA concentration with follow-up time (*P* > 0.05). Overall, the tracking coefficient of UA concentration from 3 to 9 years of age was 0.31, and was higher in boys than in girls (0.34 vs. 0.29, respectively). While the tracking coefficient of UA concentration from 5 to 9 years of age was higher (total = 0.37, boys = 0.39, girls = 0.34), these values can interpreted as showing moderate stability.

**Table 1:** Distribution of *SLC2A9* G844A and *SLC22A12* C246T variants and characteristics of the study subjects

	Unit: *n* (%) or mean (standard deviation)		
	Total	Boys	Girls
Genetic variants			
rs3825017 polymorphism			
CC	377 (61.1%)	197 (62.5%)	180 (59.6%)
CT	215 (34.9%)	107 (34.0%)	108 (35.8%)
TT	25 (4.0%)	11 (3.5%)	14 (4.6%)
rs16890979 polymorphism			
GG	614 (99.0%)	312 (98.7%)	302 (99.3%)
GA	6 (1.0%)	4 (1.3%)	2 (0.7%)
Serum uric acid concentration (μmol/L) at each FU age			
UA at 3 years of age	223.44 (46.44)	225.38 (43.98)	221.52 (48.78)
UA at 5 years of age	235.94 (42.55)	234.60 (41.55)	237.28 (43.69)
UA at 7 years of age	223.97 (55.64)	219.92 (57.03)	227.94 (54.12)
UA at 9 years of age	211.59 (48.85)	214.19 (52.04)	208.97 (45.41)
Covariates			
Birth weight (kg)	3.09 (0.59)	3.15 (0.59)	3.03 (0.58)
BMI (kg/m^2^) at 3 years of age	15.47 (1.31)	15.49 (1.33)	15.45 (1.29)
BMI (kg/m^2^) at 5 years of age	15.71 (1.67)	15.94 (1.78)	15.47 (1.53)
BMI (kg/m^2^) at 7 years of age	15.95 (2.10)	16.19 (2.18)	15.72 (2.01)
BMI (kg/m^2^) at 9 years of age	17.50 (2.58)	17.88 (2.75)	17.14 (2.35)
Maternal education level			
Graduated from high school	132 (22.2%)	64 (21.2%)	68 (23.3%)
Some college or higher	462 (77.8%)	238 (78.8%)	224 (76.7%)

*UA* uric acid, *FU* follow-up, *BMI* body mass index

**Fig. 1:**
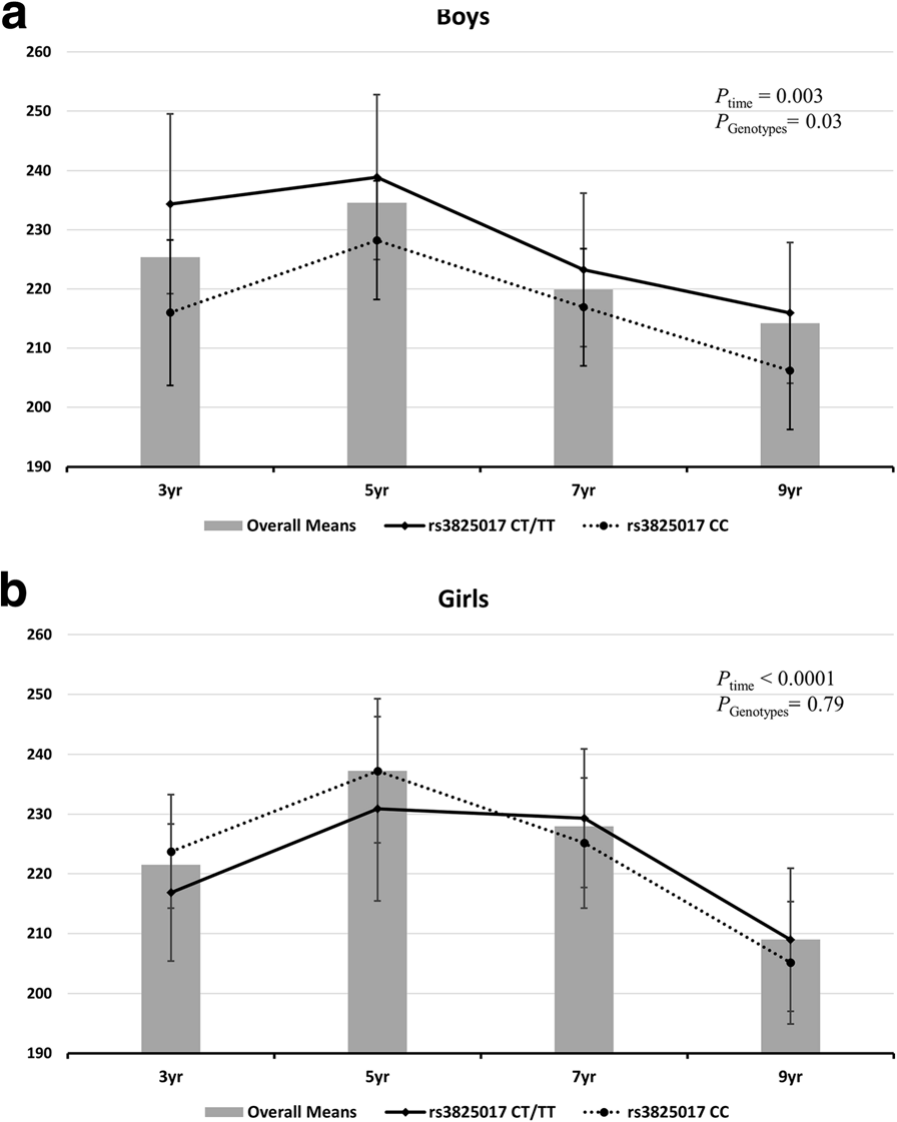
The average uric acid concentrations (μmol/L) with the dominant model of rs3825017 genotypes from 3 to 9 years of age, by sex. **a** Boys, **b** Girls The least-squared mean by genotypes (as a dominant model) at each follow-up age were obtained from a mixed model under a random intercept model with variance components structure. It was estimated while controlling for time, birth weight, maternal education level, and body mass index at each time point. Genetic variants and time were applied as categorical values to obtain the mean values for the genetic variant at each time point. The box indicates the average UA concentration, and the vertical lines show the standard error

Table 2 shows the average UA concentration for genetic models of the two polymorphisms. There was a limited ability to assess the effects of rs16890979 on UA concentration because the minor allele frequency was very low. Thus, its result was not presented in the table. The effects of rs3825017 on UA concentration were significant in boys, but not in girls. Boys with the T allele of rs3825017 had higher concentrations than their counterparts at baseline (Table 2). In boys, carriers with one or two T alleles (i.e., CT or TT) had a higher mean UA concentration than non-carriers regardless of the time of follow-up, even after adjusting for birth weight, maternal education level, time, and BMI at each follow-up point. In girls, no effects of rs3825017 on UA concentration were apparent (Fig. 1).

**Table 2:** Baseline differences in the uric acid concentrations (μmol/L) for each genetic model^a^

	Total				Boys				Girls			
	*n*	Mean	S.D.	*p*	*n*	Mean	S.D.	*p*	*n*	Mean	S.D.	*p*
Dominant model for rs3825017												
CC	222	221.58	47.98	0.37	112	219.17	45.44	0.02	110	224.02	50.52	0.40
CT + TT	151	226.02	44.40		73	234.66	40.44		78	217.94	46.64	
Recessive model for rs3825017												
CC + CT	355	223.19	46.79	0.73	178	224.02	43.97	0.05	177	222.36	49.57	0.33
TT	18	227.02	42.81		7	257.46	36.13		11	207.64	35.63	
Codominant model for rs3825017												
CC	222	221.58	47.98	0.66	112	219.17	45.44	0.02	110	224.02	50.52	0.53
CT	133	225.89	44.77		66	232.24	40.36		67	219.63	48.21	
TT	18	227.02	42.81		7	257.46	36.13		11	207.64	35.63	

*S.D* standard deviation

^a^ For the baseline differences, the results were obtained using the uric acid concentrations at 3 years of age

The longitudinal effects of rs3825017 genotype on individual UA concentration are presented in Table 3. The random intercept model showed that UA concentration at baseline varied among the children. When estimating changes in UA concentration with age, we found that concentration decreased by 4.39 μmol/L with every 2-year increase in age (*P* < 0.001). Individual UA concentrations changed at the same rate with age in the children, indicating that there were no significant effects of a random slope in time. The rs3825017 genotypes had significant effects on the changes in UA concentration in the first decade of life in boys only (Table 3). The effect of BMI on the UA concentrations was not significant in either sex. Maternal education level as a measure of socioeconomic status and birth weight had no effect on UA concentration. After adjusting for the interaction between genotypes and sex, the difference in sex was significant (*P* for interaction = 0.08). The longitudinal effects of rs16890979 were not assessed due to the very low frequency of the minor allele.

**Table 3:** Longitudinal effects of rs3825017 genotypes on individual serum uric acid concentrations

	Total			Boys			Girls		
	*β*	S.E.	*p*	*β*	S.E.	*p*	*β*	S.E.	*p*
Sex	0.48	3.37	0.89	–			–		
FU time	−4.39	1.18	< 0.001	−4.51	1.71	< 0.01	−4.06	1.65	0.01
Dominant model for rs3825017	5.13	3.44	0.14	11.98	4.94	0.02	−0.88	4.77	0.85
BMI	1.47	0.76	0.05	1.90	1.02	0.06	0.92	1.16	0.43
Birth weight	−1.50	2.92	0.61	2.57	4.08	0.53	−4.79	4.20	0.25
Maternal education	5.46	4.02	0.17	8.95	5.76	0.12	3.09	5.61	0.58

*FU* follow-up, *BMI* body mass index

The rs3825017 genotypes for the dominant model (CC and CT + TT)

The coefficients (*β*) were obtained from a mixed model under random intercept model with variance components structure

## Discussion

This study used a longitudinal approach to examine the relationship between two candidate SNPs and UA concentration in a general pediatric population. The rs3825017 genotype of *SLC22A12* had a notable effect on UA concentration in the first 10 years of life in boys. Although there was no sex difference in UA concentration, the genetic effects of rs3825017 on concentration differed by sex (*P*_genotype × sex_ = 0.08) during childhood. The tracking coefficient of UA from 3 to 9 years of age showed moderate stability.

Both *SLC22A12*, which encodes urate transporter 1 (URAT1), and *SLC2A9*, which encodes glucose transporter 9 (GLUT9), are involved in modulating urate reabsorption in the renal tubules. A meta-analysis of GWA studies identified multiple candidate loci related to UA level in European (*SLC2A9*, *ABCG2*, *SLC17A1*, *SLC22A11*, *SLC22A12*, *SLC16A9*, *GCKR*, *LRRC16A*, and *PDZK1*) and East Asian (*SLC2A9*, *ABCG2*, *SLC22A12*, and *MAF*) populations [2, 25]. Mutations in *SLC22A12* or *SLC2A9* were initially reported in Japanese patients with renal hypouricemia, although patients from various ethnic groups have subsequently been identified [7]. A study of children in the Viva La Familia Study also recently showed that the serum UA concentration was associated strongly with genetic variants in *SLC2A9* through GWA analysis [19]. Of genetic variants, the Hereditary and Phenotype Intervention (HAPI) Heart Study found that rs16890979 in *SLC2A9* was associated with UA concentrations and it accounted for 4.3% of the variance [26]. This association was also reported in studies of the Framingham and Rotterdam cohorts [5] and a Czech population [27], but there was a discrepancy in the direction of association. This might involve additional factors, such as diet, lifestyle, and different linkage disequilibrium structures [8, 27]. Although two meta-analyses reported that the dominant model of rs16890979 was associated with gout in an Asian population [9, 28], our study had limited statistical strength due to the low minor allele frequency; the minor allele frequency of rs16890979 was less than 0.02 in the populations of China and Japan, was greater than 0.29 in a European population [29], 0.23 in the Framingham Heart Study, and was 0.21 in the Rotterdam Study [5]. In addition, *SLC2A9* rs16890979 showed possible associations with gout [9] and hyperuricemia [30]. However, functional characterization of *SLC2A9* rs16890979 in an experimental validation study revealed no significant difference in the expression of GLUT9 [30]. Functional studies are likely to be important in determining the causality of disease-related SNPs.

In our study, rs3825017 in *SLC22A12* affected UA concentration in boys, but not in girls. Three other studies have examined rs3825017 [12, 15, 31]. In line with our study, one study of Korean men found an association with hyperuricemia (odds ratio = 2.29, 95% confidence interval 1.78 to 2.93), while studies from Japanese and Chinese population found no significant effect. The latter studies did not assess sex differences, which needs further study. Regarding to *SLC2A9*, several studies have suggested sex differences by genotype including rs16890979; the effects were seen more clearly in women than in men [2, 32]. The sex difference can be explained by sex hormones. Because estrogen is uricosuric [33], the differences with the rs3825017 genotype are expected to be more distinct around puberty. A recent GWA study of gout and its subtypes revealed that *HIST1H2BF-HIST1H4E*, *NIPAL1*, and *FAM35A* were novel risk loci [8]. However, detailed knowledge of the functional pathways of these genes affecting UA concentrations remains limited and further studies are required.

Although the pathological mechanism of UA is unclear, Mazzali et al. reported that rats with induced mild hyperuricemia develop mild hypertension and slightly reduced renal function [34]. In addition, injury was seen in the afferent arterioles of the renal microvasculature in the hyperuricemic rat [35, 36]. These experimental studies also suggested that these changes could be prevented by maintaining UA concentration in the normal range [1, 34, 35].

Even in children, UA had an independent effect on higher blood pressure after controlling for adiposity factors, renal function, and pubertal status [18]. A cohort study with an average 12-year follow-up also reported that the UA concentration in childhood and changes in concentration were significant predictors of blood pressure in later life [4]. Feig et al. suggested that serum UA concentration is directly correlated with blood pressure, and that hyperuricemia showed a stronger association in childhood primary hypertension than in secondary or white-coat hypertension [3]. Therefore, control of UA levels may be important to prevent disease. In our study, the tracking coefficient of UA during the 6-year follow-up period beginning at 3 years of age showed moderate stability (ICC = 0.31). The tracking coefficient of UA from 5 to 9 years of age was slightly higher (ICC = 0.37). The UA concentration in blood can vary with age, sex, and pubertal status [4, 19]. Unlike previous studies, we found no differences in UA concentration with sex, and the average UA concentration decreased with increasing age during the first decade of life. Our study examined younger subjects than those used in previous studies [4, 18, 37], and there are few prospective studies of UA using repeated measures. Thus, it is difficult to explain the difference.

To the best of our knowledge, this is the first study to assess the persistent effects of genetic variants on the UA concentration during childhood. Compared with non-carriers, boys with carriers of at least one rs3825017 T allele exhibited elevated UA concentrations. Our results support those of an earlier study conducted in Korean males [15]. Additionally, BMI was positively associated with the UA concentration during childhood, with borderline significance in boys but not in girls.

This study had several weaknesses. First, we assessed the effect of one tag SNP; other genetic variants near URAT1 may also be important. In addition, residual confounding by unmeasured factors may affect UA concentration. Conversely, our results are less likely to be influenced by unhealthy factors such as alcohol consumption and comorbidities compared to adult studies and case-control studies, because this study examined a general pediatric population. We assumed that the genetic variants were dichotomized by a dominant effect of the risk allele due to the low frequency of homozygous carriers of the risk allele. It is necessary to confirm this in a large-scale study and in studies of different ethnic groups. To verify the statistical power, we calculated the required sample size for linear mixed model with random intercept. As a result, 41 subjects per group were required to evaluate the significance of a difference in the UA concentration between the two groups (non-carriers vs carriers of at least one rs3825017 T allele), assuming that the power was 80% with α = 0.05, four repeated measurements, and mean difference − 11.3. Thus, we found that the statistical power of our study was adequate. Finally, there is a limit to generalizing our findings because the evidence is still insufficient.

## Conclusions

In summary, we estimated the stability of UA concentration over 6 years beginning in early childhood, and observed moderate stability. A single measurement of UA during childhood may have a limited ability to predict disease in later life. Through a longitudinal approach, we also found that rs3825017 genotype had a notable effect on UA concentration in boys during childhood. Our study considered only two SNPs (rs3825017 in *SLC22A12* and rs16890979 in *SLC2A9*). Therefore, further studies with extended follow-up periods are required to consider other major genetic loci associated with UA concentrations, such as glucokinase regulatory protein (*GCKR*) and *ABCG2* [2].

## Abbreviations

*ABCG*: ATP-binding cassette transporter
AIC: Akaike information criterion
BMI: Body mass index
EDTA: Ethylenediaminetetraacetic acid
*GCKR*: Glucokinase regulatory protein
GLUT9: Glucose transporter 9
GWA: Genome-wide association
ICC: Intra-class correlation
PCR: Polymerase chain reaction
*SLC22A12*: Solute carrier family 22 (organic anion/cation transporter) member 12
*SLC2A9*: Solute carrier family 2 facilitated glucose transporter member 9
SNP: Single-nucleotide polymorphism
UA: Uric acid
URAT1: Urate transporter 1
